# Evaluation of intra- and interobserver reliability in the assessment of the ‘critical trochanter angle’

**DOI:** 10.1186/s40001-020-00469-4

**Published:** 2020-12-10

**Authors:** Sebastian Serong, Moritz Schutzbach, Ivica Zovko, Marcus Jäger, Stefan Landgraeber, Marcel Haversath

**Affiliations:** 1 Department of Orthopaedics & Orthopaedic Surgery, Saarland University, Kirrberger Strasse 100, 66421 Homburg, Germany; 2 Department of Orthopaedics & Traumatology, University of Duisburg-Essen, Essen, Germany; 3 Department of Orthopaedics, Trauma and Reconstructive Surgery, St. Marien Hospital Mülheim/Chair of Orthopaedics and Trauma Surgery, University of Duisburg-Essen, Essen, Germany

**Keywords:** Critical trochanter angle, Total hip arthroplasty, Stem alignment, Intraobserver, Interobserver, Reliability

## Abstract

**Background:**

The recently described ‘critical trochanter angle’ (CTA) is a novel parameter in the preoperative risk assessment of stem malalignment in total hip arthroplasty. As its reproducibility needs to be evaluated, the given study aims to investigate intra- and interobserver reliability. It is hypothesized that both analyses justify the clinical use of the CTA.

**Methods:**

A total of 100 pelvic radiographs obtained prior to total hip arthroplasty were retrospectively reviewed by four observers with different levels of clinical experience. The CTA was measured twice by each observer at different occasions in the previously described technique. Intra- and interobserver reliability was evaluated using intraclass correlation coefficients (ICC) with confidence intervals (CI) and the Bland–Altman approach.

**Results:**

The mean CTA in both measuring sequences was 20.58° and 20.78°. The observers’ means ranged from 17.76° to 25.23°. Intraobserver reliability showed a mean difference of less than 0.5° for all four observers (95% limit of agreement: − 7.70–6.70). Intraobserver ICCs ranged from 0.92 to 0.99 (CI 0.88–0.99). For interobserver variation analysis, ICCs of 0.83 (CI 0.67–0.90) and 0.85 (CI 0.68–0.92) were calculated.

**Conclusion:**

Analyses concerning intra- and interobserver reliability in the assessment of the CTA showed ‘very good’ and ‘good’ results, respectively. In view of these findings, the use of the CTA as an additional preoperative parameter to assess the risk of intraoperative stem malalignment seems to be justified.

## Background

Preoperative planning is mandatory when performing total hip arthroplasty (THA) because it reduces the risk of inaccurate biomechanical reconstruction and may also prevent over- and undersizing of implant components [1–3]. Incorrect offset reconstruction must be avoided as it harbours the risks of alterations in leg length and postoperative gluteal insufficiency [4]. In this context, intraoperative component positioning is of the utmost importance. With regard to stem orientation in THA, several factors of influence have been identified. Amongst others, the surgical approach, implant design, femoral broach shape, the surgeon’s level of experience and the presence of deformities such as dysplasia have to be mentioned. [5–9]. Varus stem alignment in particular has been correlated to the following risk factors: low centrum collum diaphyseal angle (CCD) in coxa vara, long thigh neck anatomy, greater trochanteric height, a lower canal-flare index and distinct trochanter overhang [5, 10]. With the first description of the ‘critical trochanter angle’ (CTA), a further parameter was recently introduced for preoperative risk assessment of stem malalignment [11]. This novel geometric angle does not measure the trochanter overhang alone, but the overhang in relation to the femur shaft axis. Moreover, it is independent of the individual size of the hip. Varus stem alignment of two degrees and more had a sensitivity of 90% and a specificity of 80% in patients with a preoperative CTA of 22.75° or less [11].

As for all new parameters that may affect diagnostics, treatment or therapy outcome, the reproducibility and reliability of the CTA have to be determined in order to justify its use in everyday clinical practice. Therefore, the given study aims to investigate the intra- and interobserver reliability of the CTA.

## Methods

For retrospective analysis, 100 preoperative conventional pelvic radiographs of patients with unilateral coxarthrosis were evaluated. Radiographic evaluation confirmed osteoarthritis stage 3 and 4 according to Kellgren and Lawrence in each case [12]. All patients underwent THA at the same institution (EndoCert® certified centre of arthroplasty) between 2012 and 2015. Only collarless straight tapered stems (Corail® type) and cementless hemispheric cups via direct lateral Hardinge approach were used. Operative interventions were exclusively performed by EndoCert®-approved high volume surgeons with > 100 THAs per year.

**Fig. 1:**
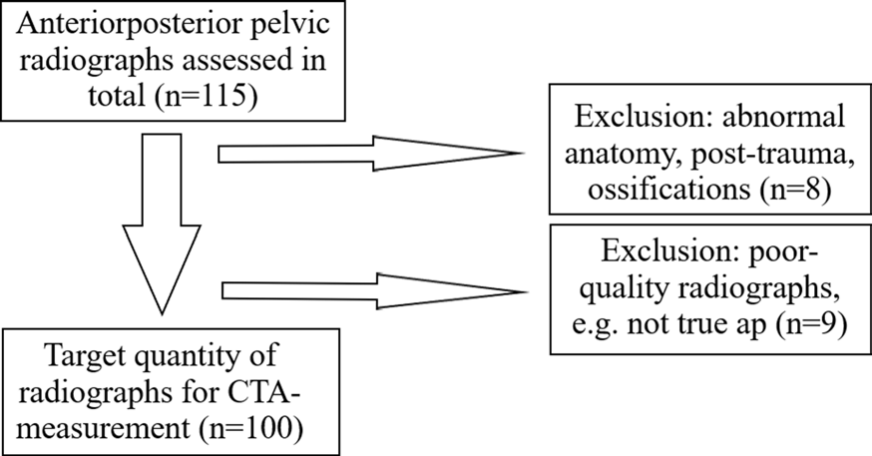
Flowchart demonstrating the inclusion/exclusion of radiographs to obtain the target quantity of *n* = 100

**Fig. 2:**
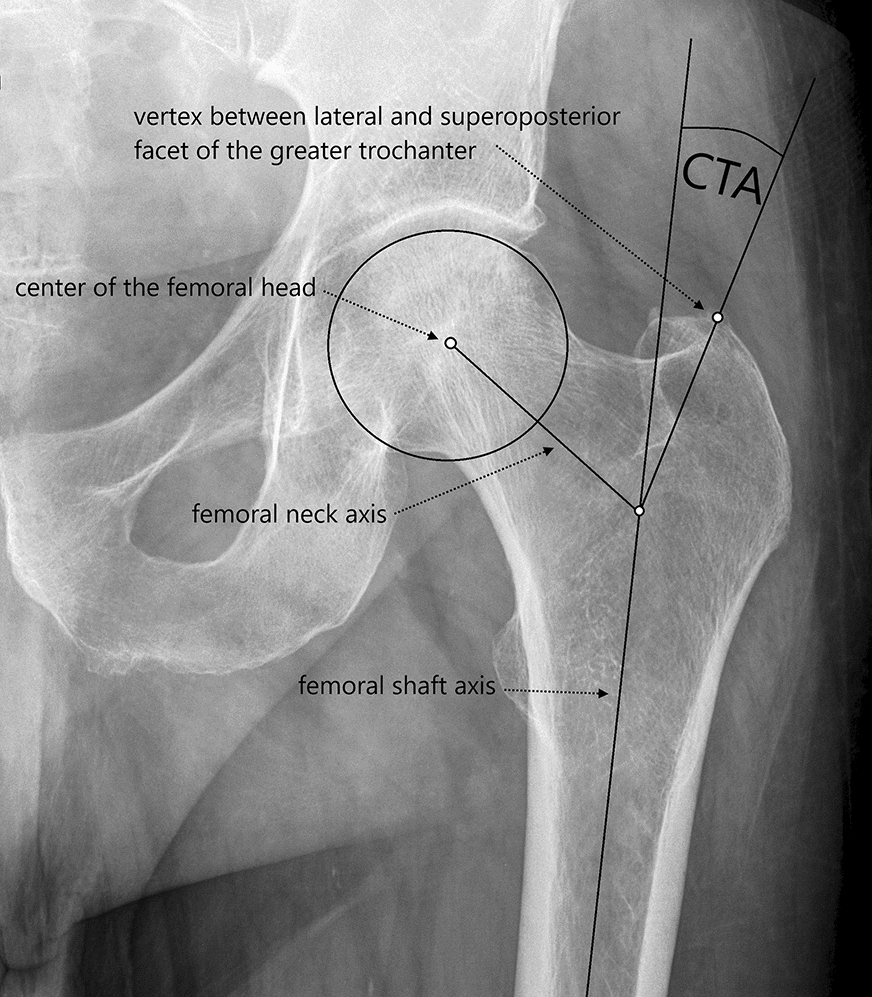
Measurement of the ‘critical trochanter angle’ (CTA) as described by Haversath et al. [11]

For evaluation in this study’s context, only standardized anteroposterior (ap) pelvic radiographs centred over the pubic symphysis were reviewed. Quality control was ensured by systematic presentation and evaluation of all performed X-ray diagnostics in weekly radiologic reviews with mandatory participation for the medical staff. Final selection for inclusion in the study was made by the first and last author (each with 10 years of experience). Radiographs showing previous fractures, abnormal head–neck anatomy or ossifications close to the trochanter were excluded (*n* = 8). Furthermore, radiographs of poor quality, e.g. no true ap-setting, were also excluded from the study (*n* = 9). In order to obtain the target quantity of 100 measurable radiographs, 115 radiographs had to be assessed in total (Fig. 1). Four of the five authors, all members of the Department of Orthopaedics & Orthopaedic Surgery of the Saarland University Medical Centre or the Department of Orthopaedics & Traumatology of the University of Duisburg-Essen, acted as observers. Two of them were tenth-year consultants [SS (observer 1) and MH (observer 2)], whereas two observers were fourth-year [MS (observer 3)] and second-year [IZ (observer 4)] residents. Due to their work on the first description of the CTA, observers 1 and 2 were familiar with performance of the measurements and instructed observers 3 and 4 in the method. Assessment of the pelvic radiographs was carried out using the mediCAD® planning software (mediCAD Hectec GmbH, Altdorf, Germany). The CTA was measured as described by Haversath et al. First, the angle crest localized at the intersection of the femoral shaft and neck axis was identified. Then, the CTA was measured between the shaft axis and leg, intersecting the vertex between the lateral and superoposterior facet of the greater trochanter (Fig. 2) [11]. The CTA was determined twice by each observer on two different occasions, though the order of the patients was changed randomly before the second measurement. Furthermore, the observers were blinded to the patients’ clinical information, to other observers’ results as well as to their own previous measurements. Additionally, they were not given any feedback between the observations.

Descriptive and comparative statistical analysis was performed using SPSS® Statistics (Version 21.0.0.0, IBM®). Normal distribution was checked by means of the Kolmogorov–Smirnov test and confirmed for all samples. The difference between the two series of each observer in their measurements was tested concerning the existence of significant differences using the one-sample *t*-test. For assessing the agreement between measurements of a continuous variable (CTA) across multiple observers the use of intraclass correlation coefficient (ICC) and Bland–Altman plot are available [13]. To evaluate intraobserver reliability, the mean difference between the two measurements of each observer was calculated and analysed concerning its relation to the 95% limits of agreement [14–16]. Visualization was realized by plotting the differences against the mean measurements as described by Bland and Altman. Intra- and interobserver reliability was tested by means of the intraclass correlation coefficient and 95% confidence interval (CI) [17, 18]. In particular, this was done using the two-way random model and absolute agreement [19].

## Results

### Intraobserver reliability

**Fig. 3:**
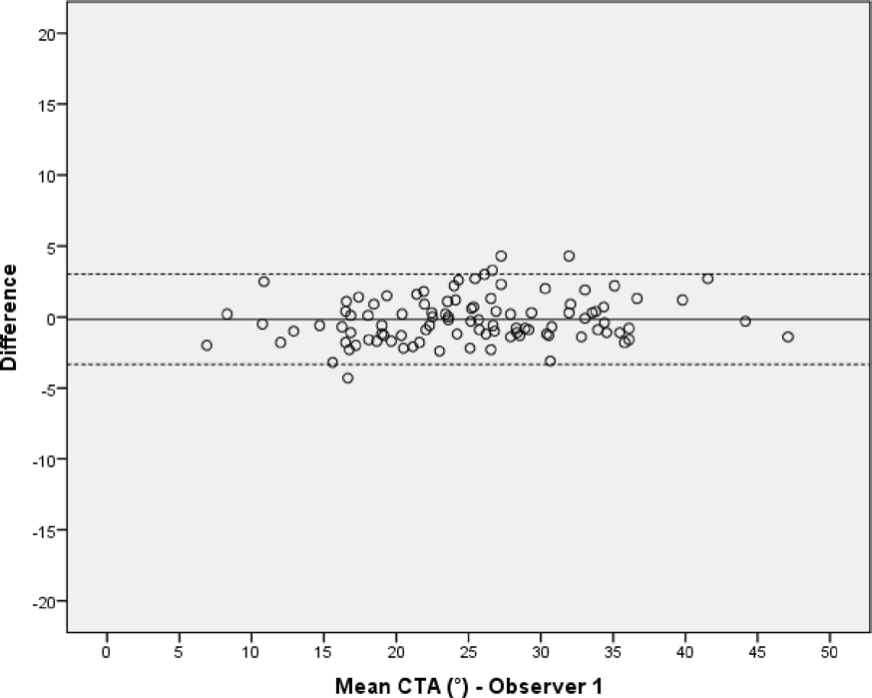
Intraobserver variation of the ‘critical trochanter angle’ (CTA) for observer 1; solid line—mean value of measurements, dotted lines—95% limits of agreement above and below the mean value

**Fig. 4:**
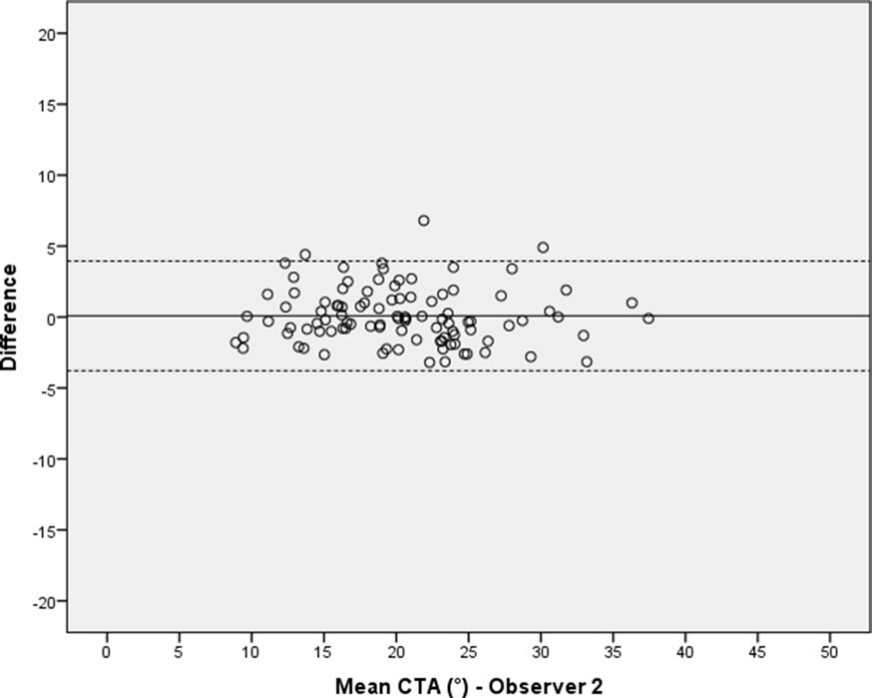
Intraobserver variation of the ‘critical trochanter angle’ (CTA) for observer 2 (for explanations see Fig. 2)

**Fig. 5:**
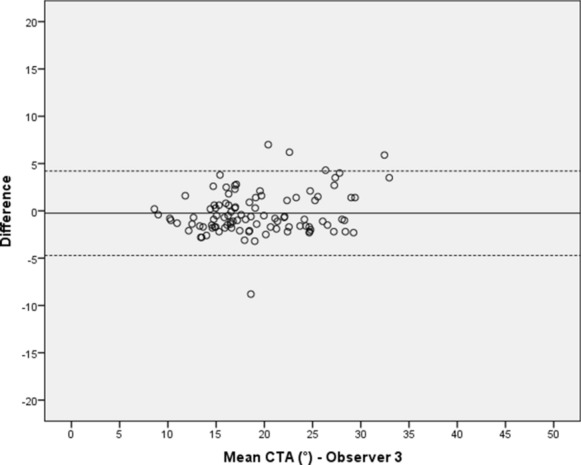
Intraobserver variation of the ‘critical trochanter angle’ (CTA) for observer 3 (for explanations see Fig. 2)

**Fig. 6:**
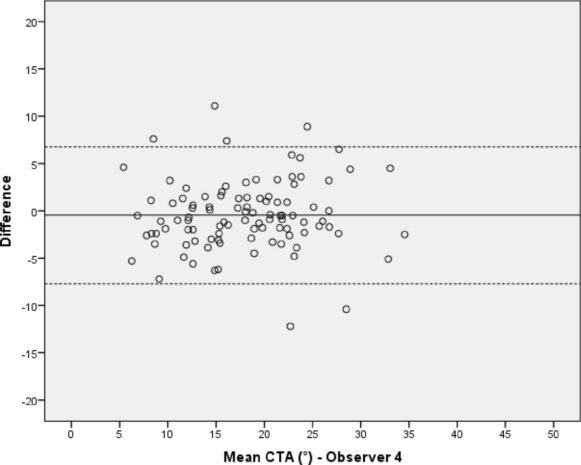
Intraobserver variation of the ‘critical trochanter angle’ (CTA) for observer 4 (for explanations see Fig. 2)

Between each observer’s first and second measuring sequence, no significant differences in the CTA values could be detected with p-values ranging from 0.21 to 0.68. The mean difference between both test series of all observers was less than 0.5° with the 95% limits of agreement ranging from -7.70° to 6.77°. Intraobservers’ ICCs ranged from 0.99 to 0.92 (Table 1). The Bland–Altman plots illustrate the proximity achieved between the two measuring sequences by plotting the differences between the two measurements of each observer against their mean values (Figs. 3, 4, 5 and 6). This shows that the measurements of observer 4 are characterized by a distinctly higher level of statistical scatter and a wider range in the 95% limits of agreement compared to the other observers.

**Table 1:** Intraobserver variation of observers 1–4 between the first and second measurement of the ‘critical trochanter angle’ (CTA)

Intraobserver variation					
Observer	Subjects	Difference mean (°) (SD)	95% limits of agreement (°)	*p* value	ICC (95% CI)
Observer 1	100	− 0.17 (1.63)	− 3.35–3.02	0.31	0.99 (0.98–0.99)
Observer 2	100	0.08 (1.97)	− 3.79–3.95	0.68	0.97 (0.96–0.98)
Observer 3	100	− 0.25 (2.28)	− 4.72–4.22	0.27	0.96 (0.93–0.97)
Observer 4	100	− 0.47 (3.69)	− 7.70–6.77	0.21	0.92 (0.88–0.94)

### Interobserver reliability

The mean CTA regarding both sequences of all four observers was 20.58° (mean min: 17.76, mean max: 25.06) for the first and 20.78° (mean min: 18.22, mean max: 25.23) for the second measurement. Interobserver correlation analysis for all four observers showed an intraclass correlation coefficient (ICC) of 0.83 (CI 0.67–0.90) for the first and an ICC of 0.85 (CI 0.68–0.92) for the second test series, respectively (Table 2).

**Table 2:** Interobserver correlation of the ‘critical trochanter angle (CTA) for both measuring sequences

Interobserver correlation				
Measuring sequence		Subjects	Mean CTA (°)	ICC (95% CI)
**1**	Observer 1	100	25.06	0.83 (0.67–0.90)
	Observer 2	100	20.34	
	Observer 3	100	19.15	
	Observer 4	100	17.76	
**2**	Observer 1	100	25.23	0.85 (0.68–0.92)
	Observer 2	100	20.26	
	Observer 3	100	19.40	
	Observer 4	100	18.22	

## Discussion

The CTA is a novel parameter which helps to evaluate the risk for intraoperative stem malpositioning in THA. According to the authors, its determination provides further and possibly more valuable information in comparison to existing parameters such as the CCD [11].

In contrast to merely focusing on correlation Bland and Altman described a statistical approach for evaluating the agreement between two different measurements of the same quantity emphasizing the importance and need for collection of replicated data by performing repeated measurements [14, 20].

In this study, significant differences between two lines of measurements by each observer could be statistically excluded, thus proving consistent data. The two measurements by each observer showed a mean difference of less than 0.5°, indicating very good repeatability. This is confirmed by the calculation of the intraobserver ICCs, which ranged from 0.92 to 0.99 for all observers and the results thus show a ‘very good’ correlation according to the interpretation recommended by Cicchetti and Koo & Li [21, 22]. The graphic visualization realized by the usage of Bland–Altman plots for all four observers demonstrate the proximity between the first and second measurement and reveal only a few outliers beyond each of the 95% limits of agreement. Additionally, a homogenous distribution of values above and below the mean difference line as well as for the mean CTA is demonstrated. Therefore, a proportional bias indicated by a trend towards above or below the mean difference or towards higher or lower CTA values in general seems to be rather unlikely [15]. Comparing the four plots with one another, the measurements of observer 4 appear to be scattered more widely. This is substantiated by a distinctly wider range of measured values and a greater standard deviation compared to the other observers. So, there is at least some indication that clinical experience plays a significant role in accurate assessment of the CTA as observer 4 was a second-year resident and the youngest participant among all observers [23, 24].

Regarding interobserver variation, mean CTA values between 17.76° and 25.23° were found. Particularly observer 1 showed a tendency towards greater values in measuring the CTA compared to the other observers. However, calculation of the intraclass correlation coefficient for interobserver reliability of the two measuring sequences presented results of 0.83 and 0.85, respectively. Again, according to the suggestions of interpretation of Cicchetti and Koo & Li, the results of the given study prove a ‘good’ (Koo & Li) to ‘very good” (Cicchetti) interobserver reliability in the assessment of the CTA [21, 22].

However, possible limitations related to the results of this study were identified. The quality of the pelvic radiographs is crucial for pursuing accurate measurements. Despite critical assessment of the radiographs used before measuring the CTA, a bias cannot be completely excluded. All observers in this study were orthopaedists or orthopaedic surgeons. Representatives from other medical disciplines, such as radiologists, might have obtained different results [23]. However, as the CTA is supposed to be a measure to estimate the risk of varus stem alignment, its clinical use is likely to be primarily performed by orthopaedic surgeons as part of preoperative planning. Finally, it must be taken into account that assessment of the CTA regarding intra- and interobserver reliability has not been done before. Therefore, as there are no similar studies with which the given results can be compared, critical evaluation of their significance is not possible. As concerns the clinical relevance of this study’s findings, it has to be pointed out that preoperative measurement of the CTA only allows a risk assessment of possible varus stem alignment due to bony characteristics. In a multifactorial setting, further parameters which are known to affect intraoperative implant positioning such as surgical approach, implant design, the surgeon’s skills and deformities still have to be paid attention to in order to achieve desirable postoperative results [5–9].

## Conclusion

The intra- and interobserver reliability of the CTA is ‘very good’ and ‘good’. Therefore, the CTA is a valuable and reproducible preoperative parameter for determining the risk for stem malalignment in THA due to bony characteristics. However, the individual observer’s level of experience in evaluating pelvic radiographs may affect the quality of CTA measurements. This is the first study to investigate the intra- and interobserver reliability in the assessment of the CTA.

## Data Availability

The datasets used and analysed during the current study are available from the corresponding author on reasonable request.

## Abbreviations

CTA: Critical trochanter angle
ICC: Intraclass correlation coefficient
CI: Confidence interval
THA: Total hip arthroplasty
CCD: Centrum collum diaphyseal angle
Ap: Anteroposterior
SD: Standard deviation
